# Surgical Outcomes of Idiopathic Epiretinal Membrane: the Gülhane Experience

**DOI:** 10.4274/tjo.00334

**Published:** 2018-04-25

**Authors:** Dorukcan Akıncıoğlu, Gökhan Özge, Murat Küçükevcilioğlu, Fazıl Cüneyt Erdurman, Ali Hakan Durukan

**Affiliations:** 1 Şanlıurfa Training and Research Hospital, Ophthalmology Clinic, Şanlıurfa, Turkey; 2 University of Health Sciences Gülhane Training and Research Hospital, Ophthalmology Clinic, Ankara, Turkey

**Keywords:** Epiretinal, volume, internal limiting membrane, correlation

## Abstract

**Objectives:**

We aimed to report our experiences and outcomes of vitreoretinal surgery in idiopathic epiretinal membrane.

**Materials and Methods:**

We retrospectively reviewed patients who underwent vitreoretinal surgery for idiopathic epiretinal membrane between January 2012 and 2014. The patients’ pre- and postoperative visual acuity, slit-lamp examination findings, and optical coherence tomography (OCT) images were evaluated.

**Results:**

Forty-five eyes of 45 patients (36% male, 64% female) were included (mean age, 69±8.2 years). Mean postoperative follow-up time was 7±4 (1-12) months. The mean preoperative logMAR best corrected visual acuity was 0.58±0.32 and postoperatively 0.40±0.31, 0.33±0.33, 0.28±0.34 respectively at 3, 6, and 12 months. All OCT parameters showed statistically significant anatomical improvement at 1, 3, 6, and 12 months. Correlation analysis showed that central macular thickness (r=0.69, p<0.05) and central macular volume (r=0.69, p<0.05) were the only parameters that had strong positive correlations with visual improvement.

**Conclusion:**

Epiretinal membrane causes heterogeneous anatomical changes in the macula for every patient. Therefore, a correlation between visual gain and changes in central macular thickness could not yet be demonstrated. We believe that central macular volume may be a better parameter for following these patients.

## Introduction

Epiretinal membrane (ERM) is a fibrocellular membrane that forms on the vitreoretinal interface due to accumulation of cells and extracellular matrix, and is often idiopathic.^1^ Besides primary idiopathic cases, ERM may also occur secondary to ocular inflammatory diseases, retinal vascular diseases, and pathologies such as retinal detachment. Unilateral ERM is more common in both primary and secondary cases, though 20-35% of patients have bilateral ERM.^2^ The literature consensus is that primary idiopathic ERM occurs more frequently in the older population; however, there is significant variation between studies in terms of prevalance.^2,3,4,5^ Prevalence rates in these studies were determined based on the presence of ERM in non-mydriatic fundus photograph. In contrast, the Beaver Dam Eye research group documented ERM by spectral domain optic coherence tomography (OCT) and reported a prevalence of 34.1% at the end of 20-year follow-up of a population with an average age of 74 years.^6^ The wide variability in reported prevalence rates may be attributable to differences in ethnicity of the study populations or medical technology utilized in the studies. Advanced age has been reported as a commonly recognized risk factor in different study groups.^7^ Despite speculation of the presence of ocular and systemic risk factors associated with ethnicity (myopia,^7^ hypermetropia^7^, smoking^5^, high education level,^7^ hypercholesterolemia,^7^ diabetes mellitus^7^), these have yet to be proven. 

ERM is a vitreoretinal interface pathology, and abnormal posterior vitreous detachment plays a key role in its development. It may develop as the result of the migration of retinal glial cells through small holes in the internal limiting membrane (ILM) formed during separation by the posterior hyaloid, and/or due to retention and transformation of some hyaloid cellular components on the retina surface during separation.^8,9^ The hypothesis that microperforations in the ILM may be responsible for the physiopathology is supported by findings of retinal pigment epithelium (RPE) cells and retinal glial cells in postoperative histopathologic ERM examination in patients with no history of trauma, laser photocoagulation, or crypexy and no previous clinical findings of retinal pathology such as tear or hole.^10^ In another study, histopathologic examination of ERM revealed the presence of vitreous hyalocytes, which supports the hypothesis that cells remaining on the retina surface following posterior vitreous detachment form a scaffold for ERM physiopathology.^9^ It is known that hyalocytes are not specific to the vitreous, but originate from bone marrow and have gone through regeneration.^11^ Therefore, although there is no scientific study proving that the hyalocytes composing the ERM structure originate from the vitreous, it is believed that ERM physiopathology involves the transformation and extracellular matrix formation of cells originating from these two mechanisms.^7^

Because idiopathic primary ERM is often a thin and transparent membrane resembling cellophane, it is also referred to as cellophane maculopathy. Cellophane maculopathy causes no traction and therefore no distortion in the neurosensorial retina or vascular structures, and is generally asymptomatic. Membrane thickening and contraction due to cellular transformation leads to distortion in the external and internal layers of retina, resulting in anatomical changes ranging from altered foveal contour to full-thickness macular hole.^12^ Patients with macular distortion generally present with complaints such as metamorphopsia, diplopia, and reduced vision. Depending on their visual complaints, the patients are either scheduled for follow-up or elective surgery. The aim of this study was to present our experiences and visual outcomes achieved with patients who underwent surgery for visual complaints and idiopathic ERM.

## Materials and Methods

**Figure 1:**
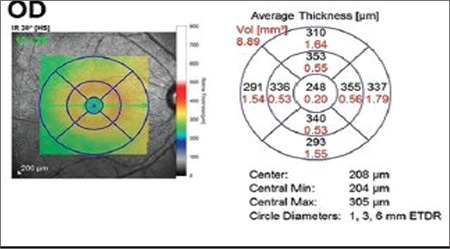
The thickness and volume values for the zones shown in the circle diagram were used as optical coherence tomography parameters ETDR: Early Treatment Diabetic Retinopathy Study, OD: Right eye

The records of patients who underwent vitreoretinal surgery in our clinic due to idiopathic ERM between January 2012 and June 2014 were examined retrospectively. The study was conducted in accordance with the principles of the Declaration of Helsinki and was approved by the local ethics committee. Pre- and postoperatively all patients underwent a detailed ophthalmologic examination including best corrected visual acuity (BCVA), anterior and posterior segment examination, and OCT. Standard triple sclerotomy followed by standard phacoemulsification surgery, intraocular lens implantation, and vitrectomy were performed for patients with nuclear sclerosis; standard triple sclerotomy and pars plana vitrectomy (PPV) was performed in patients without cataract. All surgeries were done using a 23-gauge Constellation^®^ vision system (Alcon; Fort Worth, Texas). In the PPV, cut and flow rates were adjusted according to the patient’s condition during core vitrectomy and removal of the vitreous base and hyaloid. A standard cut rate was not used in the surgeries, but a lower rate was preferred for vitrectomy and a higher rate was preferred for shaving. Following core vitrectomy, posterior hyaloid removal assisted by 0.1 mL (4 mg) triamcinolone acetonide (Kenacort-A; 40 mg/mL; Bristol-Myers Squibb, Princeton, NJ, USA), and vitreous base removal, the ILM was stained with brilliant blue or a dual dye and peeled from the area between the major vascular arcades. Examination and OCT (Spectralis, Heidelberg Engineering, Heidelberg, Germany) findings at postoperative 1, 3, 6, and 12 months were evaluated. All imaging in the study was done by the same experienced technician using the same equipment, which is important in terms of data standardization for retrospective research. When evaluating the macula in patients with heterogeneous ERM-related macular changes, in our clinic we prefer raster scanning using a macular cube scanning protocol consisting of 6x6 mm square fovea-centered sections. Images obtained from the patients included in the study were used to prepare a macular map using a 1-, 3-, and 6-mm Early Treatment Diabetic Retinopathy Study grid (Figure 1).

Functional success was evaluated based on BCVA increase (logMAR). Evaluation of anatomic success was based on pre- and postoperative OCT measurements of central macular thickness (CMT) and the maximum, average, and minimum thicknesses in the central 1-mm zone and thickness/volume in the central 3-mm and 6-mm areas.

### Statistical Analysis

Descriptive and statistical analyses of the data were done using SPSS version 21.0 software. Changes in visual acuity and OCT measurements were evaluated using paired-samples t-test. Correlation between these changes was assessed using Spearman’s correlation coefficient for the non-normally distributed variables central maximum thickness, central 3-mm thickness, central 3-mm volume, and central 6-mm volume. Pearson’s correlation coefficient was used for the other normally distributed variables.

## Results

**Figure 2:**
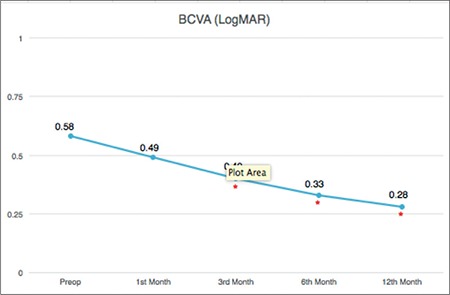
Best corrected visual acuity was significantly increased at postoperative 3, 6, and 12 months compared to preoperative levels

Of the 45 patients included in the study, 16 (36%) were males and 29 (64%) were females, and the mean age was 69±8.2 years. The mean postoperative follow-up period was 7±4 (1-12) months. BCVA was worse than 0.70 logMAR in 48.9% of the patients preoperatively but only 6.6% of the patients postoperatively. Mean BCVA of all patients was 0.58±0.32 preoperatively, 0.40±0.31 at postoperative 3 months, 0.33±0.33 at 6 months, and 0.28±0.34 at 12 months. The increase in BCVA was significant at 3, 6, and 12 months (Figure 2; Table 1). Postoperatively, 39 patients (86.8%) had a gain in visual acuity of at least two lines, while 4 patients (8.8%) showed no change. The lack of change was attributed to cataract in two of those patients and cystoid macular edema in the other two. Visual acuity declined in 2 (4.4%) cases, one due to retinal detachment in the third month and the other due to intense cataract development. 

**Figure 3:**
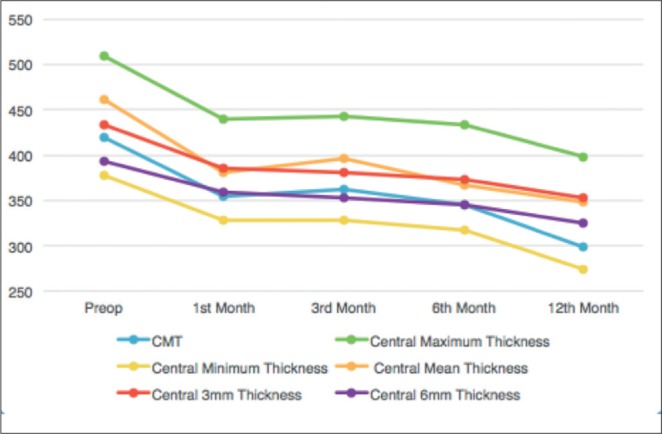
Postoperative change in optical coherence tomography parameters related to macular thickness

**Figure 4:**
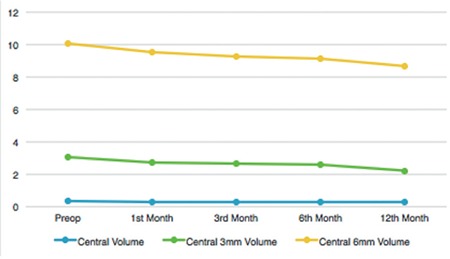
Postoperative change in the optical coherence tomography parameters related to macular volume

Comparison of pre- and postoperative OCT measurements of CMT, central maximum, central minimum, central average, and central 3-mm, and central 6mm thickness and volume values demonstrated significant anatomic recovery at 1, 3, 6, and 12 months (Figure 3 and 4; Tables 2 and 3).

Correlation analysis of increased BCVA and changes in OCT values revealed statistically significant, strong positive correlations between visual gain and central average thickness (r=0.69, p<0.05) and central volume change (r=0.69, p<0.05) (Table 4). CMT, central minimum thickness, and central 3-mm volume values were negatively correlated with visual gain, but the relationships were not statistically significant. The other parameters had weak but nonsignificant positive correlation.

## Discussion

In the Blue Mountains Eye study, of 245 eyes with baseline ERM, progression was observed in 29%, regression in 26%, and no change in 39% of the eyes at 5-year follow-up.^12^ Only 20% of the cases classified as cellophane maculopathy in that study showed progression. In our clinic, we also inform patients diagnosed with cellophane maculopathy about the symptomatology and invite them for regular follow-up. Findings of irreversible photoreceptor damage in OCT are associated with poor postoperative visual prognosis, and thus early surgeries afford better visual prognosis.^13^ Therefore, we recommend early surgical treatment before photoreceptor damage is detected in OCT. Cataract progression accelerates in phakic eyes following ERM surgery.^14^ Rahman and Stephenson^15^ also reported that patients who had early surgery, especially combined procedures including cataract removal, experienced faster postoperative visual rehabilitation with better visual gains. Among our patients, 14 (31%) underwent combined procedures, and there were no differences between the pseudophakic and combined surgery groups in terms of postoperative visual acuity or amount of visual improvement. In a 10-year retrospective analysis, Dawson et al.^16^ observed no significant differences between the combined surgery and pseudophakic groups in terms of final visual acuity levels or pre- and postoperative complications. However, they reported that the patients with higher preoperative visual acuity had higher final visual acuity and those with lower preoperative visual acuity had more significant increases in visual acuity. The most common early complication after combined surgery is increased intraocular pressure.^17^ The most common late complication is posterior capsule opacification.^18^ We did not encounter elevated intraocular pressure or posterior capsule opacification severe enough to cause visual symptoms in any of our patients postoperatively. One of the shortcomings of this retrospective study is that we have no data regarding progression of posterior capsule opacification. In their prospective study, Ahfat et al.^18^ reported a high rate of posterior capsule opacification (42.1%). ILM peeling in the same session is recommended because cells remaining after ERM removal can form a new scaffold on the ILM and lead to recurrent ERM.^19^ None of the patients in our study who underwent routine ILM peeling assisted by dual dye experienced recurrence. 

Surgical decisions for ERM patients should be based primarily on their OCT changes and symptoms such as metamorphopsia or micropsia instead of preoperative visual acuity because there is a known association between reduced visual acuity and photoreceptor damage; therefore, these patients have poor visual prognosis.^13^ Functional gains are evaluated based on visual acuity and improvement of patients’ preoperative subjective complaints. Anatomic gains are followed with CMT and foveal contour on OCT imaging. In a study by Güngel et al.^20^, no correlation was found between postoperative visual gains and reduced macular thickness. Other studies investigating this relationship also revealed no correlation between macular thickness and visual gains.^21,22^ The present study was planned with the belief that the lack of correlation between reduced macular thickness and functional gains is due to the heterogeneous macular distortion caused by the ERM and that there may be a correlation with changes in central macular volume as opposed to thickness, and we found that the postoperative reduction in macular volume in the central 1-mm area was correlated with functional visual gains.

## Conclusion

Although ERM formation may occur on different parts of the retina due to different etiologies, visual complaints are generally caused by ERM that develops over the macula. Macular ERMs can cause symptoms proportionate to the degree of distortion and subsequent photoreceptor damage that they cause. CMT is not a reliable source of information about heterogeneous tissue distortion; therefore, monitoring changes in central volume over follow-up can give more accurate results. Furthermore, in prospective studies, patients with different central volumes may be grouped preoperatively for a comparison of postoperative gains. This may allow the planning of surgical treatment for patients who have reached a predetermined central volume value before photoreceptor damage and the onset of visual complaints, thus enabling better outcomes.

## Figures and Tables

**Table 1:**
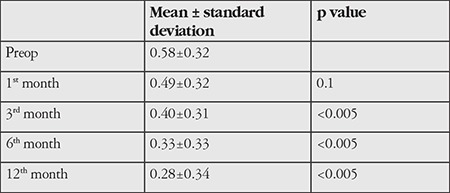
Preoperative and postoperative best corrected visual acuity values (logMAR)

**Table 2:**
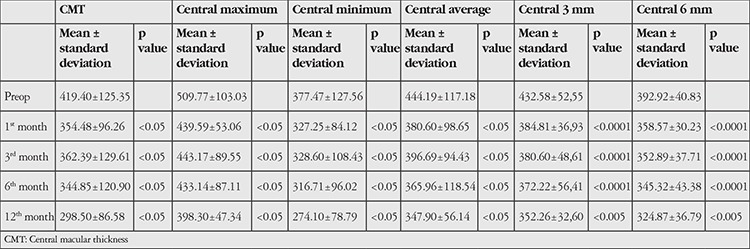
Comparison of preoperative and postoperative thickness parameters on optical coherence tomography

**Table 3:**
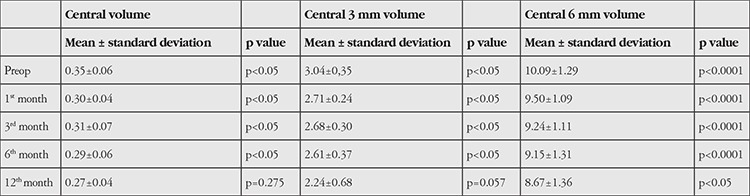
Comparison of preoperative and postoperative volume parameters on optical coherence tomography

**Table 4:**
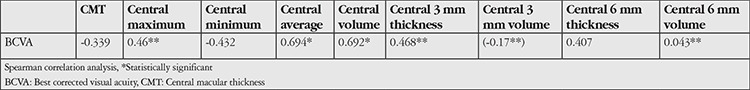
Correlation between postoperative optical coherence tomography parameters and best corrected visual acuity levels
